# The Imperative to Share Clinical Study Reports: Recommendations from the Tamiflu Experience

**DOI:** 10.1371/journal.pmed.1001201

**Published:** 2012-04-10

**Authors:** Peter Doshi, Tom Jefferson, Chris Del Mar

**Affiliations:** 1 Johns Hopkins University School of Medicine, Baltimore, Maryland, United States of America; 2 The Cochrane Collaboration, Roma, Italy; 3 Centre for Research in Evidence-Based Practice, Bond University, Gold Coast, Australia

## Abstract

Peter Doshi and colleagues describe their experience trying and failing to access clinical study reports from the manufacturer of Tamiflu and challenge industry to defend their current position of RCT data secrecy.

**Summary Points**

- Systematic reviews of published randomized clinical trials (RCTs) are considered the gold standard source of synthesized evidence for interventions, but their conclusions are vulnerable to distortion when trial sponsors have strong interests that might benefit from suppressing or promoting selected data.
- More reliable evidence synthesis would result from systematic reviewing of clinical study reports—standardized documents representing the most complete record of the planning, execution, and results of clinical trials, which are submitted by industry to government drug regulators.
- Unfortunately, industry and regulators have historically treated clinical study reports as confidential documents, impeding additional scrutiny by independent researchers.
- We propose clinical study reports become available to such scrutiny, and describe one manufacturer's unconvincing reasons for refusing to provide us access to full clinical study reports. We challenge industry to either provide open access to clinical study reports or publically defend their current position of RCT data secrecy.

Regulatory approval of new drugs is assumed to reflect a judgment that a medication's benefits outweigh its harms. Despite this, controversy over approved drugs is common. If sales can be considered a proxy for utility, the controversies surrounding even the most successful drugs (such as blockbuster drugs) seem all the more paradoxical, and have revealed the extent to which the success of many drugs has been driven by sophisticated marketing rather than verifiable evidence [1],[2]. But even among institutions that aim to provide the least biased, objective assessments of a drug's effects, determining “the truth” can be extremely difficult.

Consider the case of the influenza antiviral Tamiflu (oseltamivir). Prior to the global outbreak of H1N1 influenza in 2009, the United States alone had stockpiled nearly US$1.5 billion dollars worth of the antiviral [3]. As the only drug in its class (neuraminidase inhibitors) available in oral form, Tamiflu was heralded as the key pharmacologic intervention for use during the early days of an influenza pandemic when a vaccine was yet to be produced. It would cut hospitalizations and save lives, said the US Department of Health and Human Services (HHS) [4]. The Advisory Committee on Immunization Practices (ACIP, the group the US Centers for Disease Control and Prevention [CDC] uses to form national influenza control policy) said it would reduce the chances of developing complications from influenza [5]. So, too, did the Australian Therapeutic Goods Administration [6] and the European Medicines Agency (EMA) [7].

Most (perhaps all) of these claims can be traced back to a single source: a meta-analysis published in 2003 that combined ten randomized clinical trials conducted during the late 1990s by the manufacturer prior to US registration of the drug [8]. This analysis, conducted by Kaiser and colleagues, proposed that oseltamivir treatment of influenza reduced both secondary complications and hospital admission. In contrast, the Food and Drug Administration (FDA), which approved Tamiflu in 1999 and was aware of these same clinical trials, concluded that Tamiflu had *not* been shown to reduce complications, and required an explicit statement in the drug's label to that effect [9]. FDA even cited Roche, Tamiflu's manufacturer, for violation of the law for claims made to the contrary [10].

Nor did the FDA approve an indication for Tamiflu in the prevention of transmission of influenza [9],[11]. This assumption was at the heart of the World Health Organization's (WHO) proposed plan to suppress an emergent pandemic through mass prophylaxis [12]. While the WHO recently added Tamiflu to its Essential Medicines list, if FDA is right, the drug's effectiveness may be no better than aspirin or acetaminophen (paracetemol). The FDA has never clarified the many discrepancies in claims made over the effects of Tamiflu. Although it may have limited approval indications accordingly, the FDA has never challenged the US HHS or the US CDC for making far more ambitious claims. This means that critical analysis by an independent group such as a Cochrane review group is essential.

But which data should be used? In updating our Cochrane review of neuraminidase inhibitors, we have become convinced that the answer lies in analyzing clinical study reports rather than the traditional published trials appearing in biomedical journals [13]. Clinical study reports contain the same information as journal papers (usually in standardized sections, including an introduction, methods, results, and conclusion [14]), but have far more detail: the study protocol, analysis plan, numerous tables, listings, and figures, among others. They are far larger (hundreds or thousands of pages), and represent the most complete synthesis of the planning, execution, and results of a clinical trial. Journal publications of clinical trials may generate media attention [2], propel researchers' careers, and generate some journals a revenue stream [15]. However, when regulators decide whether to register a new drug in a manufacturer's application, they review the trial's clinical study report.

In 2010, we began our Cochrane review update using clinical study reports rather than published papers [16]. We obtained some sections of these clinical study reports for the ten trials appearing in the Kaiser 2003 meta-analysis from Tamiflu's manufacturer, Roche—around 3,200 pages in total. In 2011, we obtained additional sections of clinical study reports for Tamiflu through a Freedom of Information request to the EMA, amounting to tens of thousands of pages. While extensive and detailed, it is important to note that what we have obtained is just a subset of the full clinical study reports in Roche's possession. Nonetheless, Box 1 provides a list of details we have already discovered—and would have never discovered without access to these documents. This information has turned our understanding of the drug's effects on its head. Other drugs for which previously unpublished, detailed clinical trial data have radically changed public knowledge of safety and efficacy include Avandia, Neurontin, and Vioxx (Table 1).

**Box 1. What Is Missed without Access to Tamiflu Clinical Study Reports**

1. Knowledge of the total denominator. (How many trials were conducted on this drug that might fit the systematic review inclusion criteria?) [13]
2. Realization that serious adverse events (SAEs) occurred in trials for which SAEs were not reported in published papers [13].
3. Understanding what happened in some trials that were published 10 years after completion [43].
4. Vital details of trials (content and toxicity profile of placebos, mode of action of drug, description and temporality of adverse events) [11].
5. Authorship is not consistent with published papers [44] (although, if a review's inclusion criteria include clinical study reports, authorship is not an issue, as the responsibility is clearly the manufacturer's).
6. Rationale for alternatively classifying outcomes such as pneumonia as a complication or an adverse event [16].
7. Ability to know whether key subgroup analysis (influenza-infected subjects) is valid [11].
8. Assessment of validity of previously released information on the drug (articles, reviews, conferences, media, etc.).
9. Realization that Roche's claim of Tamiflu's mode of action [45] appears inconsistent with the evidence from trials [11],[46].

**Table 1:** Different sources of data for the uncovering of failures in reporting of safety and effectiveness of some examples of new drugs.

Drug (Trade Name; Manufacturer)	Brief History	Type and Magnitude of the Problem	How the Problem Was Discovered
**Rosiglitazone** (Avandia; GSK)	FDA approval granted in 1999 for use in diabetes. EMA did not approve, initially, over concerns about lack of evidence of efficacy, but granted approval in 2000. In 2001, FDA issued new warnings over concerns about cardiovascular risks (especially acute myocardial infarction). In a separate lawsuit in 2004, GSK agreed to publicly post online details of all its clinical trials. In 2010, the EMA suspended rosiglitazone. The company is dealing with 10,000 to 20,000 cases of litigation in the United States alone.	The primary trials had weaknesses of the methods, including excessive loss to follow-up, and over-emphasis on proxy outcome measures (blood glucose levels rather than mortality and morbidity outcomes) [27]–[29].	2005–2007 series of meta-analyses increasingly suggest harms, especially cardiovascular.Public disclosure of unpublished study results was critical to uncovering the evidence of harm [30].
**Gabapentin** (Neurontin; Parke-Davis, a Pfizer subsidiary)	FDA approval was granted in 1994 for a narrow indication: adjunctive treatment for epilepsy (partial seizures). The company was accused of using aggressive marketing using systematic methods, including key opinion leaders (KOLs), to promote off-label use [31]–[33]. Most sales were for off-label use. In 2002, FDA approval was extended to post-herpetic neuralgia. However, in May 2004, Pfizer agreed to pay a US$240 million criminal fine in the United States for illegally promoting off-label use [34].	Delay in publishing company sponsored trials, such as a 1998 Pfizer-funded study showing lack of efficacy for bipolar disease, in which publication was delayed for two years [34].	An internal whistleblower drew attention to the problem, which resulted in legal action taken by US authorities [34],[35].
**Rofecoxib** (Vioxx; Merck)	FDA approval granted in 1999. In 2000, the VIGOR study was published, apparently demonstrating superiority over non selective inhibition [36]. Data claimed to have been excluded in a critical editorial [37]. In 2004, Merck withdrew the drug. Subsequently, Merck has been defending against thousands of liability legal actions [38] after educational events had been attempted to declare rofecoxib was safe [39].	Unexpected and unanticipated cardiovascular events (principally, myocardial infarction).A trial of rofecoxib conducted to check protection against colon polyps established an increased cardiovascular disease (CVD) risk [39].	Concern from a regulator (FDA) that there might be increased CVD events caused by the drug. Trial proposed, but not conducted [39].Clinical study reports indirectly important in uncovering the scandal.
**Oseltamivir** (Tamiflu; Roche)	FDA approval granted in 1999. Sales surged following concern over avian and pandemic influenza in 2005 and 2009, leading to massive government stockpiles worldwide. Numerous governmental bodies assumed the drug would reduce the complications of influenza, and hospitalizations, based on a Roche-supported meta-analysis of some efficacy trials [8]. This claim was challenged by our 2009 Cochrane review update in response to criticism [40].	There was a public call for individual patient data, already provided confidentially to regulators, to be made available for public scrutiny [41]. Roche agreed to release “full study reports”, but (as discussed in this article) later offered multiple reasons for not providing these data.	The latest Cochrane update relied on clinical study reports obtained from regulators (freedom of information requests), reviews, and other documents released by regulators and leaked sources. The manufacturer provided incomplete reports for some trials. Our review has led to the detection of numerous reporting biases and fundamental problems in trial design, and we have concluded that previous effectiveness claims were not supported by the available evidence.

## An Urgent Call for a Debate on the Ethics of Data Secrecy

Taken together, these experiences suggest that any attempt at reliable evidence synthesis must begin with clinical study reports. Yet we are aware of only three other groups of independent researchers conducting a systematic review based entirely (or mostly) on these and other regulatory documents [17]–[19]. We can think of two major reasons this might be. First, outside of regulatory agencies, few researchers have ever heard of clinical study reports. Second, clinical study reports are massive in size and difficult to obtain, traditionally shared only with regulators. The first problem seems tractable, but gaining better access to manufacturers' clinical study reports requires shifting the status quo from a default position of confidentiality to one of disclosure.

In the mid-20th century, regulatory agencies became increasingly responsible to the public for ensuring the safety and effectiveness of approved medicines. This rise paralleled an increase in the number and complexity of clinical trials. Both in the United States and Europe, manufacturers would come to submit trial data to regulators with the assurance that authorities would treat all trade secret data as confidential. Even following passage of the 1966 Freedom of Information Act (FOIA), the FDA maintained that safety and effectiveness test data were not subject to the FOIA [20],[21]. One sign things may be changing came in November 2010, when the EMA announced its intention to make all industry clinical study reports about a drug publicly available as a matter of standard practice after reaching a regulatory decision [22],[23]. The EMA has also already improved its handling of data under Freedom of Information requests. But as we learned was true for Tamiflu, regulators may not be in possession of full trial reports of all studies on a given intervention, implying the necessity of obtaining reports from manufacturers [11]. But at present, industry seems extremely reluctant to make its clinical study reports freely available.

In addition to clinical study reports, we also need access to regulatory information. By the very nature of their professional mandate, regulators may conduct some of the most thorough evaluations of a trial program, and efforts like ours aimed at up-to-date evidence synthesis are seriously deprived without access to their reports. While the FDA has increasingly published its reviews, memos, and other correspondence on its Drugs@FDA website (http://www.accessdata.fda.gov/scripts/cder/drugsatfda/), and additional documents are accessible through FOIA requests, the process can be very time-consuming, taking months or even years. Moreover, regulatory agencies' lack of public inventories of their documentary holdings complicates the retrieval of information. Ideally, we would also have details of the regulators' deliberations, which can serve as signposts to important issues that need investigating.

In December 2009, after we voiced serious concerns in the *BMJ* about Tamiflu's alleged ability to reduce complications [24], Roche wrote that it was “very happy to have its data reviewed by appropriate authorities or individuals,” and publicly pledged to release “full study reports” for ten trials “within the coming days” [25]. But despite extensive correspondence over the next year and a half, Roche refused to provide any more than portions of the clinical study reports for the ten Kaiser studies (Table 2) and no reports for any of the additional Tamiflu trials we had subsequently identified and requested (Table 3). Reasons for refusing to share the full reports on Tamiflu kept changing, and none seemed credible (Tables 2 and 3).

**Table 2:** Roche's reasons for not sharing its clinical study reports and the authors' response (for 10 trials in Kaiser meta-analysis [8]).

Roche's Basis for Reluctance or Refusal to Share Data	Response
“Unfortunately we are unable to send you the data requested as a similar meta analysis is currently commencing with which there are concerns your request may conflict. We have been approached by an independent expert influenza group and as part of their meta analysis we have provided access to Roche's study reports.” (Oct. 8, 2009)	It is unclear why another group of independent researchers would prevent Roche from sharing the same data with our group.
Cochrane reviewers were “unwilling to enter into the [confidentiality] agreement with Roche.” (Dec. 8, 2009)	The terms of Roche's proposed contract were unacceptable to us. We declined to sign for two reasons: 1) all data disclosed under the contract were to be regarded as confidential; and 2) signing the contract would also require us “not to disclose … the existence and terms of this Agreement”. We judged that the requirement to keep all data, and the confidentiality agreement itself, secret would interfere with our explicit aim of openly and transparently systematically reviewing the trial data and accounting for their provenance.
Roche says that it was “under the impression that you [Cochrane] were also satisfied with its provision based on our correspondence earlier this year (March 2010).” (June 1, 2010)	We did not immediately realize that what Roche had provided was incomplete. Irrespective of whether we had at one point seemed “satisfied,” Roche had not delivered what it publicly promised in the *BMJ* on Dec. 8, 2009: “full study reports will also be made available on a password-protected site within the coming days to physicians and scientists undertaking legitimate analyses.”
“… around 3,200 pages of information have already been provided by Roche for review by your group and the scientific community.” (Aug. 20, 2010)	What is important is completeness, and 3,200 pages is a fraction of the full study reports for the ten Kaiser trials Roche promised to make available.
“Roche undertook this action [release of 3,200 pages] to demonstrate our complete confidence in the data and our commitment to transparency to the degree to which patient confidentiality, data exclusivity and the protection of intellectual property allow.” (Aug. 20, 2010)	This implies that release of the promised-but-never-released data would impinge on “patient confidentiality, data exclusivity and the protection of intellectual property”. This does not seem to apply to many elements of clinical study reports (e.g., the trial protocol and reporting analysis plan), and it is unclear why personal data could not be anonymized.
“The amount of data already made accessible to the scientific community through our actions extends beyond what is generally provided to any third party in the absence of a confidentiality agreement.” (Aug. 20, 2010)	It is irrelevant what is “generally provided”. What is relevant is what was promised and the need for public disclosure of clinical study reports.
“Over the last few months, we have witnessed a number of developments which raise concerns that certain members of Cochrane Group involved with the review of the neuraminidase inhibitors are unlikely to approach the review with the independence that is both necessary and justified. Amongst others, this includes incorrect statements concerning Roche/Tamiflu made during a recent official enquiry into the response to last year's pandemic by a member of this Cochrane Review Group. Roche intends to follow up separately to clarify this issue. We also note with concern that certain investigators, who the Cochrane Group is proposing will carry out the planned review, have previously published articles covering Tamiflu which we believe lack the appropriate scientific rigor and objectivity.” (Aug. 20, 2010)	Despite Roche's promise and our request for specifics, Roche never responded directly.
“We noted in our correspondence to the BMJ in December of last year our concern that the first requests for data to assist in your review did not come from the Cochrane Group, but from the media apparently trying to obtain data following discussions with the Cochrane Review Group. This raised serious questions regarding the motivation for the review from the outset. We note that in subsequent correspondence regarding your next planned review you have copied a number of journalists when responding to emails sent by Roche staff.” (Aug. 20, 2010)	Our view is that Tamiflu is a global public health drug and the media have a legitimate reason for helping independent reviewers obtain data, which includes being informed of our efforts to do so.
Cochrane reviewers have been provided with “all the trial data [they] require …” (Jan. 14, 2011)	We disagree. First, it is up to us to decide what we require. Second, we now know that what was provided was not enough. For example, Roche did not provide us with the trial protocols and full amendment history.
“You have all the detail you need to undertake a review and so we have decided not to supply any more detailed information. We do not believe the requested detail to be necessary for the purposes of a review of neuraminidase inhibitors.” (April 26, 2011)	We have still not received what was promised in December 2009, and we know that what we have received is deficient.
“It is the role of Global Regulatory Authorities to review detailed information of medicines when assessing benefit/risk. This has occurred, and continues to occur with Tamiflu, as with all other medicines, through regular license updates.” (April 26, 2011)	Independent researchers such as the Cochrane Collaboration share the goal of assessing benefit/risk, and require all details necessary to competently perform this function.
“Roche has made full clinical study data available to health authorities around the world for their review as part of the licensing process.” [42] (Jan. 20, 2012)	Roche may have made full clinical study data “available” but that does not mean they “provided” all regulators with full clinical study data. For at least 15 Tamiflu trials, Roche did not provide the European regulator (EMA) with full study reports, apparently because EMA did not expressly request the complete clinical study reports. (Correspondence with EMA, May 24 and Jul. 20, 2011)

**Table 3:** Roche's reasons for not sharing its clinical study reports and the authors' response (for other Tamiflu trials).

Roche's Basis for Not Sharing Data	Response
“With regards your recent request for additional data, we are taking the request very seriously and as such must assess this within the wider organization particularly with respect to our obligations around patient and physician confidentiality, legality and feasibility” (July 24, 2010)	We agree that “patient and physician confidentiality, legality and feasibility” are valid concerns. However, Roche has never provided details.
With respect to clinical study reports for additional studies beyond the original ten meta-analyzed by Kaiser: “we request that you submit your full analysis plan for review by Roche and the scientific/medical community.” (Aug. 20, 2010)	This is a reasonable request. However, we shared our systematic review protocol [16] with Roche on Dec. 11, 2010, following peer-review and publication online-first on the cochrane.org website (the usual Cochrane systematic review practice).
“Concerning access to the reports beyond the Kaiser analysis … many are pharmacokinetic studies conducted in healthy volunteers and so are unlikely to be useful in a meta analysis. The studies in prevention and in children are published in peer reviewed publications, or in summary form on www.rochetrials.com.” (Jan. 14, 2011)	What Roche has directed us to is insufficient (peer-reviewed publications and the extremely compressed summaries of trials on http://www.rochetrials.com) as the topic of our review is unpublished data, most prominently clinical study reports.
“The long list of studies you sent are either published in peer reviewed publications, in summary form on www.rochetrials.com, halted early, or ongoing.” (Feb. 14, 2011)	Roche continues to imply the evidentiary equivalence between unpublished clinical study reports and extremely compressed summary formats such as journal publications—a position we reject. We have repeatedly stated and shown evidence (in our study protocol and elsewhere [13],[16]) that reliable evidence synthesis requires access to clinical study reports.

There are strong ethical arguments for ensuring that all clinical study reports are publicly accessible. It is the public who take and pay for approved drugs, and therefore the public should have access to complete information about those drugs. We should also not lose sight of the fact that clinical trials are experiments conducted on humans that carry an assumption of contributing to medical knowledge. Non-disclosure of complete trial results undermines the philanthropy of human participants and sets back the pursuit of knowledge.

Potentially valid reasons for restricting the full public release of clinical study reports include: ensuring patient confidentiality (although this could be remedied with redaction); commercial secrets (although these should be clear after drugs have been registered, and we found no commercially sensitive information in the clinical study reports received from EMA with minimal redactions); and finally, industry concerns over adversaries' malicious “cherry picking” over large datasets (which could be reduced by requiring the prospective registration of research protocols). None appear insurmountable.

With the EMA's stated intentions on far wider data disclosure, we hope the debate may soon shift from one of whether to release regulatory data to the specifics of doing so. But until these policies go into effect—and perhaps even after they do—most drugs on the market will remain those approved in an era in which regulators protected industry's data [26]. It is therefore vital to know where industry stands. If drug companies have legitimate reasons for maintaining the status quo of treating all of their data as trade secret, we have yet to hear them. We are all ears.

## Supporting Information

Alternative Language Summary Points S1 **Translation of the Summary Points into Russian by Vasiliy Vlassov.**
(DOC)

Alternative Language Summary Points S2 **Translation of the Summary Points into Italian by Tom Jefferson.**
(DOCX)

Alternative Language Summary Points S3 **Translation of the Summary Points into Japanese by Yuko Hara and Yasuyuki Kato.**
(DOC)

Alternative Language Summary Points S4 **Translation of the Summary Points into Croatian by Mirjana Huić.**
(DOC)

Alternative Language Summary Points S5 **Translation of the Summary Points into Danish by Andreas Lundh.**
(DOC)

Alternative Language Summary Points S6 **Translation of the Summary Points into German by Gerd Antes.**
(DOC)

Alternative Language Summary Points S7 **Translation of the Summary Points into Spanish by Juan Andres Leon.**
(DOC)

Alternative Language Summary Points S8 **Translation of the Summary Points into Chinese by Han-Pu Tung.**
(DOC)

## Abbreviations

ACIP: Advisory Committee on Immunization Practices
CDC: US Centers for Disease Control and Prevention
EMA: European Medicines Agency
FDA: US Food and Drug Administration
HHS: US Department of Health and Human Services
WHO: World Health Organization
