# Supplementary material for: The Imperative to Share Clinical Study Reports: Recommendations from the Tamiflu Experience
Source: PLoS Med. 2012 Apr 10;9(4):e1001201. doi: 10.1371/journal.pmed.1001201 (PMC3323511; doi:10.1371/journal.pmed.1001201)
Supplement: Alternative Language Summary Points S1 — Translation of the Summary Points into Russian by Vasiliy Vlassov. (DOC) [file pmed.1001201.s001.doc]

# The imperative to share clinical study reports: recommendations from the Tamiflu experience

Peter Doshi

Johns Hopkins University School of Medicine, Baltimore, Maryland, USA

Tom Jefferson

The Cochrane Collaboration, Roma, Italy

Chris Del Mar

Centre for Research in Evidence-Based Practice, Bond University, Gold Coast, Australia

Corresponding author: Peter Doshi <pnd@jhu.edu>

## Summary Points

- Systematic reviews of published randomized clinical trials (RCTs) are considered the gold standard source of synthesized evidence for interventions, but their conclusions are vulnerable to distortion when trial sponsors have strong interests (commercial or otherwise) that might benefit from suppressing or promoting selected data.
- More reliable evidence synthesis would result from systematic reviewing of clinical study reports—standardized documents representing the most complete record of the planning, execution, and results of clinical trials, which are submitted by industry to government drug regulators.
- Unfortunately, industry and regulators have historically treated clinical study reports as confidential documents, impeding additional scrutiny by independent researchers.
- We propose clinical study reports become available to such scrutiny, and describe one manufacturer’s unconvincing reasons for refusing to provide us access to full clinical study reports. We challenge industry to either provide open access to clinical study reports or publically defend their current position of RCT data secrecy.

# Необходимость обнародовать отчеты о клинических исследованиях: рекомендации на основе опыта с Тамифлю

Питер Доши

Johns Hopkins University School of Medicine, Baltimore, Maryland, USA

Том Джефферсон

The Cochrane Collaboration, Roma, Italy

Крис Дель Мар

Centre for Research in Evidence-Based Practice, Bond University, Gold Coast, Australia

Автор для переписки: Peter Doshi <pnd@jhu.edu>

## Основные выводы

- Систематические обзоры опубликованных рандомизированных клинических испытаний (РКИ) рассматриваются как золотой стандарт синтеза доказательств об эффектах вмешательств, но их выводы подвержены искажениям, если спонсоры испытаний имеют сильную заинтересованность (коммерческую или иную) в том, чтобы ограничить распространение некоторых данных.
- Более надежный синтез доказательств возможен при систематическом обзоре отчетов о клинических испытаниях – стандартизованных документов, представляющих собою наиболее полное описание планирования, выполнения и результатов клинических исследований – представляемых производителями в правительственные регуляторные агентства.
- К несчастью, индустрия и регуляторы исторически относятся к отчетам о клинических исследованиях как к конфиденциальным документам, блокируя возможности дополнительного анализа независимым экспертам.
- Мы предлагаем, чтобы отчеты о клинических исследованиях стали доступны для такого анализа и описываем неубедительные аргументы одного производителя в пользу отказа в доступе к полным отчетам о клиническом исследовании. Мы вызываем индустрию или предоставить открытый доступ к отчетам о клинических исследованиях, или публично обосновать их нынешнюю позицию засекречивания данных об РКИ.

Translated by Vasiliy Vlassov.
