# Supplementary material for: The Imperative to Share Clinical Study Reports: Recommendations from the Tamiflu Experience
Source: PLoS Med. 2012 Apr 10;9(4):e1001201. doi: 10.1371/journal.pmed.1001201 (PMC3323511; doi:10.1371/journal.pmed.1001201)
Supplement: Alternative Language Summary Points S2 — Translation of the Summary Points into Italian by Tom Jefferson. (DOCX) [file pmed.1001201.s002.docx]

# The imperative to share clinical study reports: recommendations from the Tamiflu experience

Peter Doshi

Johns Hopkins University School of Medicine, Baltimore, Maryland, USA

Tom Jefferson

The Cochrane Collaboration, Roma, Italy

Chris Del Mar

Centre for Research in Evidence-Based Practice, Bond University, Gold Coast, Australia

Corresponding author: Peter Doshi <pnd@jhu.edu>

## Summary Points

- Systematic reviews of published randomized clinical trials (RCTs) are considered the gold standard source of synthesized evidence for interventions, but their conclusions are vulnerable to distortion when trial sponsors have strong interests (commercial or otherwise) that might benefit from suppressing or promoting selected data.
- More reliable evidence synthesis would result from systematic reviewing of clinical study reports—standardized documents representing the most complete record of the planning, execution, and results of clinical trials, which are submitted by industry to government drug regulators.
- Unfortunately, industry and regulators have historically treated clinical study reports as confidential documents, impeding additional scrutiny by independent researchers.
- We propose clinical study reports become available to such scrutiny, and describe one manufacturer’s unconvincing reasons for refusing to provide us access to full clinical study reports. We challenge industry to either provide open access to clinical study reports or publically defend their current position of RCT data secrecy.

# La necessità assoluta della condivisione dei rapporti degli studi clinici: raccomandazioni dall’esperienza con il Tamiflu

Peter Doshi

Johns Hopkins University School of Medicine, Baltimore, Maryland, USA

Tom Jefferson

The Cochrane Collaboration, Roma, Italia

Chris Del Mar

Centre for Research in Evidence-Based Practice, Bond University, Gold Coast, Australia

Corrispondenza a: Peter Doshi <pnd@jhu.edu>

## Punti Riassuntivi

- Le revisioni sistematiche di studi controllati randomizzati (RCTs) sono ritenute la miglior fonte di prove sintetizzate sugli effetti degli interventi sanitari. Tuttavia le loro conclusioni possono subire condizionamenti o “distorsioni” qualora gli interessi (commerciali e non) delle aziende sponsor potrebbero trarre vantaggio dalla soppressione o da una selezione opportunistica dei dati.
- Una sintesi più affidabile di prove cliniche sono le revisioni sistematiche di rapporti di studi clinici. Si tratta di documenti preparati secondo una metodologia standardizzata e molto rigorosa che contengono informazioni complete e dettagliate circa la pianificazione, esecuzione e i risultati dei trial clinici utilizzati dalle aziende produttrici come documentazione ai fini di approvazione da parte delle agenzie regolatorie.
- Purtroppo sia l’industria sia le agenzie governative hanno tradizionalmente considerato i rapporti di studi clinici alla stregua di documenti riservati, impedendone così un esame da parte di ricercatori indipendenti.
- Proponiamo che i rapporti di studi clinici diventino liberamente accessibili alla consultazione dei ricercatori indipendenti e descriviamo le ragioni non convincenti addotte da un produttore per negare l’accesso a rapporti completi. Sfidiamo l’industria a fornire libero accesso ai dati integrali derivanti dalle sperimentazioni o a giustificare pubblicamente la propria posizione sulla segretezza dei dati provenienti dagli RCT.

Translated by Tom Jefferson (author)
