# Supplementary material for: The Imperative to Share Clinical Study Reports: Recommendations from the Tamiflu Experience
Source: PLoS Med. 2012 Apr 10;9(4):e1001201. doi: 10.1371/journal.pmed.1001201 (PMC3323511; doi:10.1371/journal.pmed.1001201)
Supplement: Alternative Language Summary Points S3 — Translation of the Summary Points into Japanese by Yuko Hara and Yasuyuki Kato. (DOC) [file pmed.1001201.s003.doc]

# The imperative to share clinical study reports: recommendations from the Tamiflu experience

Peter Doshi

Johns Hopkins University School of Medicine, Baltimore, Maryland, USA

Tom Jefferson

The Cochrane Collaboration, Roma, Italy

Chris Del Mar

Centre for Research in Evidence-Based Practice, Bond University, Gold Coast, Australia

Corresponding author: Peter Doshi <pnd@jhu.edu>

## Summary Points

- Systematic reviews of published randomized clinical trials (RCTs) are considered the gold standard source of synthesized evidence for interventions, but their conclusions are vulnerable to distortion when trial sponsors have strong interests (commercial or otherwise) that might benefit from suppressing or promoting selected data.
- More reliable evidence synthesis would result from systematic reviewing of clinical study reports—standardized documents representing the most complete record of the planning, execution, and results of clinical trials, which are submitted by industry to government drug regulators.
- Unfortunately, industry and regulators have historically treated clinical study reports as confidential documents, impeding additional scrutiny by independent researchers.
- We propose clinical study reports become available to such scrutiny, and describe one manufacturer’s unconvincing reasons for refusing to provide us access to full clinical study reports. We challenge industry to either provide open access to clinical study reports or publically defend their current position of RCT data secrecy.

# 治験総括報告書共有の要請: タミフルの事例からの提言

ピーター　ドーシ

ジョンズ・ホプキンス大学医学部、アメリカ合衆国　メリーランド州ボルチモア

トム　ジェファーソン

コクラン共同計画、イタリア　ローマ

クリス　デル・マール

ボンド大学根拠に基づいた医療研究センター、オーストラリア　ゴールド・コースト

論文に関する連絡先: ピーター　ドーシ <pnd@jhu.edu>

## 要点

- 無作為化臨床試験（RCTs）のシステマティック・レビューは、医学的介入についてのエビデンス（科学的根拠）を明らかにするゴールド・スタンダードと考えられている。一方で、臨床試験を行う出資者が特定のデータを隠蔽または強調したりすることで得られる（営利目的または他の）利益に強い関心を示す場合、試験結果が歪められやすい。
- 治験総括報告書とは、臨床試験計画、実施、及び結果について最も完全に記録した、標準化された文書であり、産業界により医薬品規制当局へ提出されるものである。こうした治験総括報告書についてシステマティック・レビューを行うことで、より信頼性の高いエビデンスを明らかにできると我々は考える。
- 残念ながら、これまで、治験総括報告書は、産業界と医薬品規制当局により機密文書として扱われており、独立した外部研究者による更なる精査を妨げてきた。
- 我々は、そうした精査を可能にするため、治験総括報告書を公開することを提言する。また、我々が完全な治験総括報告書を入手できなかったことに関し、一製造業者の説得力に欠けた理由付けを叙述する。我々は、産業界に対し、治験総括報告書の公開を求める。さもなければ無作為化臨床試験（RCTs）データを非公開とする産業界の現行の立場を公に説明するよう申し立てる。

Translated by Yuko Hara and Yasuyuki Kato.
