# Supplementary material for: The Imperative to Share Clinical Study Reports: Recommendations from the Tamiflu Experience
Source: PLoS Med. 2012 Apr 10;9(4):e1001201. doi: 10.1371/journal.pmed.1001201 (PMC3323511; doi:10.1371/journal.pmed.1001201)
Supplement: Alternative Language Summary Points S4 — Translation of the Summary Points into Croatian by Mirjana Huić. (DOC) [file pmed.1001201.s004.doc]

# The imperative to share clinical study reports: recommendations from the Tamiflu experience

Nužnost dostupnosti izvješća kliničkih ispitivanja: preporuke temeljene na Tamiflu iskustvu

Peter Doshi

Johns Hopkins University School of Medicine, Baltimore, Maryland, USA

Tom Jefferson

The Cochrane Collaboration, Roma, Italy

Chris Del Mar

Centre for Research in Evidence-Based Practice, Bond University, Gold Coast, Australia

Corresponding author: Peter Doshi <pnd@jhu.edu>

Autor za dopisivanje:

## Summary Points

Ključne točke sažetka

- Systematic reviews of published randomized clinical trials (RCTs) are considered the gold standard source of synthesized evidence for interventions, but their conclusions are vulnerable to distortion when trial sponsors have strong interests (commercial or otherwise) that might benefit from suppressing or promoting selected data.
- Sustavni pregledi objavljenih randomiziranih kontroliranih ispitivanja (engl. randomized controlled trials, RCTs) predstavljaju zlatni standard sažimanja (sinteze) dokaza učinaka intervencije, ali njihovi zaključci su podložni iskrivljenju kad sponzor ispitivanja ima snažan interes (financijski ili neki drugi) koji može donijeti korist od zatajivanja ili promicanja određenih podataka.
- More reliable evidence synthesis would result from systematic reviewing of clinical study reports—standardized documents representing the most complete record of the planning, execution, and results of clinical trials, which are submitted by industry to government drug regulators.
- Sažimanje dokaza postaje puno vjerodostojnije sustavnim pregledom izvješća kliničkih ispitivanja - standardiziranih dokumenata koji predstavljaju najobuhvatniji zapis planiranja, provođenja i rezultata kliničkih ispitivanja, koja dostavljaju proizvođači lijekova ili medicinskih proizvoda državnim regulatornim ustanovama.
- Unfortunately, industry and regulators have historically treated clinical study reports as confidential documents, impeding additional scrutiny by independent researchers.
- Nažalost, proizvođači lijekova ili medicinskih proizvoda kao i regulatorne ustanove već povijesno smatraju izvješća kliničkih ispitivanja povjerljivim dokumentima, onemogućavajući time dodatno temeljito proučavanje neovisnim istraživačima.
- We propose clinical study reports become available to such scrutiny, and describe one manufacturer’s unconvincing reasons for refusing to provide us access to full clinical study reports. We challenge industry to either provide open access to clinical study reports or publically defend their current position of RCT data secrecy.
- Preporučamo da izvješća kliničkih ispitivanja postanu dostupna takvom neovisnom pogledu i prikazujemo proizvođačeve neuvjerljive razloge za odbijanje uvida u potpuna izvješća kliničkih ispitivanja. Zahtijevamo od proizvođača slobodan javni uvid izvješćima kliničkih ispitivanja ili njihovu javnu obranu tajnosti podataka randomiziranih kontroliranih ispitivanja.

**Text in Croatia language:**

Nužnost dostupnosti izvješća kliničkih ispitivanja: preporuke temeljene na Tamiflu iskustvu

Peter Doshi

Johns Hopkins University School of Medicine, Baltimore, Maryland, USA

Tom Jefferson

The Cochrane Collaboration, Roma, Italy

Chris Del Mar

Centre for Research in Evidence-Based Practice, Bond University, Gold Coast, Australia

Autor za dopisivanje: Peter Doshi [pnd@jhu.edu](mailto:pnd@jhu.edu)

Ključne točke sažetka

- Sustavni pregledi objavljenih randomiziranih kontroliranih ispitivanja (engl. randomized controlled trials, RCTs) predstavljaju zlatni standard sažimanja (sinteze) dokaza učinaka intervencije, ali njihovi zaključci su podložni iskrivljenju kad sponzor ispitivanja ima snažan interes (financijski ili neki drugi) koji može donijeti korist od zatajivanja ili promicanja određenih podataka.
- Sažimanje dokaza postaje puno vjerodostojnije sustavnim pregledom izvješća kliničkih ispitivanja - standardiziranih dokumenata koji predstavljaju najobuhvatniji zapis planiranja, provođenja i rezultata kliničkih ispitivanja, koja dostavljaju proizvođači lijekova ili medicinskih proizvoda državnim regulatornim ustanovama.
- Nažalost, proizvođači lijekova ili medicinskih proizvoda kao i regulatorne ustanove već povijesno smatraju izvješća kliničkih ispitivanja povjerljivim dokumentima, onemogućavajući time dodatno temeljito proučavanje neovisnim istraživačima.
- Preporučamo da izvješća kliničkih ispitivanja postanu dostupna takvom neovisnom pogledu i prikazujemo proizvođačeve neuvjerljive razloge za odbijanje uvida u potpuna izvješća kliničkih ispitivanja. Zahtijevamo od proizvođača slobodan javni uvid izvješćima kliničkih ispitivanja ili njihovu javnu obranu tajnosti podataka randomiziranih kontroliranih ispitivanja.

Translation by Mirjana Huić.
