# Supplementary material for: The Imperative to Share Clinical Study Reports: Recommendations from the Tamiflu Experience
Source: PLoS Med. 2012 Apr 10;9(4):e1001201. doi: 10.1371/journal.pmed.1001201 (PMC3323511; doi:10.1371/journal.pmed.1001201)
Supplement: Alternative Language Summary Points S5 — Translation of the Summary Points into Danish by Andreas Lundh. (DOC) [file pmed.1001201.s005.doc]

# The imperative to share clinical study reports: recommendations from the Tamiflu experience

Peter Doshi

Johns Hopkins University School of Medicine, Baltimore, Maryland, USA

Tom Jefferson

The Cochrane Collaboration, Roma, Italy

Chris Del Mar

Centre for Research in Evidence-Based Practice, Bond University, Gold Coast, Australia

Corresponding author: Peter Doshi <pnd@jhu.edu>

## Summary Points

- Systematic reviews of published randomized clinical trials (RCTs) are considered the gold standard source of synthesized evidence for interventions, but their conclusions are vulnerable to distortion when trial sponsors have strong interests (commercial or otherwise) that might benefit from suppressing or promoting selected data.
- More reliable evidence synthesis would result from systematic reviewing of clinical study reports—standardized documents representing the most complete record of the planning, execution, and results of clinical trials, which are submitted by industry to government drug regulators.
- Unfortunately, industry and regulators have historically treated clinical study reports as confidential documents, impeding additional scrutiny by independent researchers.
- We propose clinical study reports become available to such scrutiny, and describe one manufacturer’s unconvincing reasons for refusing to provide us access to full clinical study reports. We challenge industry to either provide open access to clinical study reports or publically defend their current position of RCT data secrecy.

**Nødvendigheden af at dele clinical study reports: anbefalinger baseret på erfaringerne fra Tamiflu**

Peter Doshi
Johns Hopkins University School of Medicine, Baltimore, Maryland, USA

Tom Jefferson
The Cochrane Collaboration, Roma, Italien

Chris Del Mar
Centre for Research in Evidence-Based Practice, Bond University, Gold Coast, Australien

Korrespondance: Peter Doshi <pnd@jhu.edu>

**Sammenfatning**

- Systematiske oversigtsartikler, baseret på publicerede lodtrækningsforsøg, betragtes som hovedkilden til sammenfatning af evidens for medicinske interventioner. Men deres konklusioner kan fordrejes, når forsøgssponsorerne har stærke interesser (kommercielle eller andre), der kan drage fordel af, at udvalgte data undertrykkes eller fremhæves.
- Sammenfatning af evidens vil være mere pålidelig, hvis den er baseret på systematiske oversigtsartikler af clinical study reports - standardiserede dokumenter, der repræsenterer den mest omfattende dokumentation for planlægning, udførelse og resultater af lodtrækningsforsøg, som bliver indsendt af medicinalindustrien til lægemiddelmyndighederne.
- Desværre har industrien og myndighederne hidtil behandlet clinical study reports som fortrolige dokumenter, hvilket hæmmer yderligere undersøgelser foretaget af uafhængige forskere.
- Vi foreslår at clinical study reports bliver tilgængelige for sådanne undersøgelser, og vi beskriver en producents lidet overbevisende begrundelser for at nægte os fuldstændig adgang til clinical study reports. Vi opfordrer industrien til enten at give fri adgang (open access) til clinical study reports eller offentligt forsvare deres nuværende holdning om hemmeligholdelse af data fra lodtrækningsforsøg.

Translation by Andreas Lundh
