# Supplementary material for: The Imperative to Share Clinical Study Reports: Recommendations from the Tamiflu Experience
Source: PLoS Med. 2012 Apr 10;9(4):e1001201. doi: 10.1371/journal.pmed.1001201 (PMC3323511; doi:10.1371/journal.pmed.1001201)
Supplement: Alternative Language Summary Points S6 — Translation of the Summary Points into German by Gerd Antes. (DOC) [file pmed.1001201.s006.doc]

# The imperative to share clinical study reports: recommendations from the Tamiflu experience

Peter Doshi

Johns Hopkins University School of Medicine, Baltimore, Maryland, USA

Tom Jefferson

The Cochrane Collaboration, Roma, Italy

Chris Del Mar

Centre for Research in Evidence-Based Practice, Bond University, Gold Coast, Australia

Corresponding author: Peter Doshi <pnd@jhu.edu>

## Summary Points

- Systematic reviews of published randomized clinical trials (RCTs) are considered the gold standard source of synthesized evidence for interventions, but their conclusions are vulnerable to distortion when trial sponsors have strong interests (commercial or otherwise) that might benefit from suppressing or promoting selected data.
- More reliable evidence synthesis would result from systematic reviewing of clinical study reports—standardized documents representing the most complete record of the planning, execution, and results of clinical trials, which are submitted by industry to government drug regulators.
- Unfortunately, industry and regulators have historically treated clinical study reports as confidential documents, impeding additional scrutiny by independent researchers.
- We propose clinical study reports become available to such scrutiny, and describe one manufacturer’s unconvincing reasons for refusing to provide us access to full clinical study reports. We challenge industry to either provide open access to clinical study reports or publically defend their current position of RCT data secrecy.
- Systematische Übersichtsarbeiten von publizierten randomisierten klinischen Studien (RCTs) werden als Goldstandard für die zusammengefasste Evidenz für die Bewertung von Interventionen betrachtet. Ihre Schlussfolgerungen sind jedoch anfällig für Verzerrungen, wenn Studiensponsoren ausgeprägte (kommerziell oder aus anderen Gründen) Interessen haben, für die die Unterdrückung oder Bevorzugung von selektionierten Daten dienlich ist.
- Zuverlässigere Evidenzsynthesen wären das Ergebnis der systematischen Begutachtung der Reports von klinischen Studien – also der standardisierten Dokumente, die das vollständigste Bild von der Planung und Durchführung sowie von den Ergebnissen zeigen, wie sie von der Industrie an die Zulassungsbehörden eingereicht werden.
- Leider behandeln Industrie und Behörden aus historischen Gründen diese Berichte von klinischen Studien als vertrauliche Dokumente, wodurch die zusätzliche Prüfung durch unabhängige Wissenschaftler verhindert wird.
- Wir schlagen vor, Berichte von klinischen Studien solchen Überprüfungen zugänglich zu machen, und beschreiben die wenig überzeugenden Gründe eines Herstellers, mit denen uns der Zugang zu den vollständigen Studienberichten verweigert wurde. Wir fordern die Industrie auf, entweder den offenen Zugang zu den Studienberichten zu gewähren oder die gegenwärtige Position der Geheimhaltung von RCT – Daten öffentlich zu verteidigen.

Translation by Gerd Antes
