# Supplementary material for: The Imperative to Share Clinical Study Reports: Recommendations from the Tamiflu Experience
Source: PLoS Med. 2012 Apr 10;9(4):e1001201. doi: 10.1371/journal.pmed.1001201 (PMC3323511; doi:10.1371/journal.pmed.1001201)
Supplement: Alternative Language Summary Points S7 — Translation of the Summary Points into Spanish by Juan Andres Leon. (DOC) [file pmed.1001201.s007.doc]

# The imperative to share clinical study reports: recommendations from the Tamiflu experience

Peter Doshi

Johns Hopkins University School of Medicine, Baltimore, Maryland, USA

Tom Jefferson

The Cochrane Collaboration, Roma, Italy

Chris Del Mar

Centre for Research in Evidence-Based Practice, Bond University, Gold Coast, Australia

Corresponding author: Peter Doshi <pnd@jhu.edu>

## Summary Points

- Systematic reviews of published randomized clinical trials (RCTs) are considered the gold standard source of synthesized evidence for interventions, but their conclusions are vulnerable to distortion when trial sponsors have strong interests (commercial or otherwise) that might benefit from suppressing or promoting selected data.
- More reliable evidence synthesis would result from systematic reviewing of clinical study reports—standardized documents representing the most complete record of the planning, execution, and results of clinical trials, which are submitted by industry to government drug regulators.
- Unfortunately, industry and regulators have historically treated clinical study reports as confidential documents, impeding additional scrutiny by independent researchers.
- We propose clinical study reports become available to such scrutiny, and describe one manufacturer’s unconvincing reasons for refusing to provide us access to full clinical study reports. We challenge industry to either provide open access to clinical study reports or publically defend their current position of RCT data secrecy.

# La necesidad de compartir los reportes de estudios clínicos: recomendaciones a partir de la experiencia con Tamiflu.

Peter Doshi

Johns Hopkins University School of Medicine, Baltimore, Maryland, USA

Tom Jefferson

The Cochrane Collaboration, Roma, Italy

Chris Del Mar

Centre for Research in Evidence-Based Practice, Bond University, Gold Coast, Australia

Corresponding author: Peter Doshi <pnd@jhu.edu>

## Sumario:

- Las revisiones sistemáticas de estudios médicos aleatorios (RCTs) se consideran el máximo estándar como fuente de evidencia sintentizada para intervenciones, pero las conclusiones de éstas son vulnerables de ser distorsionadas cuando los patrocinadores tienen grandes intereses (comerciales u otros) que puedan beneficiarse de la supresión o promoción de información selecta.
- Una síntesis más confiable de la evidencia resultaría de la revisión sistemática de los reportes de estudios clínicos –documentos estandarizados que representan un registro completo de la planeación, ejecución y resultados de los reportes clínicos, y que son presentados por la industria a los reguladores gubernamentales.
- Desafortunadamente, la industria y los reguladores han tratado históricamente los reportes de estudios clínicos como documentos confidenciales, lo que impide su escrutinio por investigadores independientes.
- Nosotros proponemos que los reportes de estudios clínicos se hagan disponibles para este tipo de escrutinio, y describimos una de las razones poco convincentes dadas por un fabricante para negarnos acceso completo a los estudios clínicos completos. Le pedimos a la industria que nos permita acceso a los reportes de estudios clínicos, o que defienda públicamente su posición actual respecto a la confidencialidad de los reportes de estudios clínicos.

Translation by Juan Andres Leon
