# Supplementary material for: The Imperative to Share Clinical Study Reports: Recommendations from the Tamiflu Experience
Source: PLoS Med. 2012 Apr 10;9(4):e1001201. doi: 10.1371/journal.pmed.1001201 (PMC3323511; doi:10.1371/journal.pmed.1001201)
Supplement: Alternative Language Summary Points S8 — Translation of the Summary Points into Chinese by Han-Pu Tung. (DOC) [file pmed.1001201.s008.doc]

# The imperative to share clinical study reports: recommendations from the Tamiflu experience

Peter Doshi

Johns Hopkins University School of Medicine, Baltimore, Maryland, USA

Tom Jefferson

The Cochrane Collaboration, Roma, Italy

Chris Del Mar

Centre for Research in Evidence-Based Practice, Bond University, Gold Coast, Australia

Corresponding author: Peter Doshi <pnd@jhu.edu>

## Summary Points

- Systematic reviews of published randomized clinical trials (RCTs) are considered the gold standard source of synthesized evidence for interventions, but their conclusions are vulnerable to distortion when trial sponsors have strong interests (commercial or otherwise) that might benefit from suppressing or promoting selected data.
- More reliable evidence synthesis would result from systematic reviewing of clinical study reports—standardized documents representing the most complete record of the planning, execution, and results of clinical trials, which are submitted by industry to government drug regulators.
- Unfortunately, industry and regulators have historically treated clinical study reports as confidential documents, impeding additional scrutiny by independent researchers.
- We propose clinical study reports become available to such scrutiny, and describe one manufacturer’s unconvincing reasons for refusing to provide us access to full clinical study reports. We challenge industry to either provide open access to clinical study reports or publically defend their current position of RCT data secrecy

# 论分享临床研究报告之必要性：基于克流感经验之刍议

Peter Doshi

Johns Hopkins University School of Medicine, Baltimore, Maryland, USA

Tom Jefferson

The Cochrane Collaboration, Roma, Italy

Chris Del Mar

Centre for Research in Evidence-Based Practice, Bond University, Gold Coast, Australia

Corresponding author: Peter Doshi <pnd@jhu.edu>

## 要点；

- 虽然针对已出版的随机临床试验（RCTs）进行系统性的检验被视为取得介入医疗合成式证据的标准来源，但是，当试验出资人基于（不论是商业性的或是否其他）的目的而可能得益于隐抑或宣扬特定数据时，这些检验的结论却容易受到操弄。
- 更可靠的证据合成应来自对临床研究报告的系统性检视。这些由业界呈交给政府药品监管者的标准化文件乃是对机临床试验的筹备，执行与结果最完整的记录。
- 可惜的是，业界与监管者长期以来将临床研究报告视为机密文件，阻碍了独立研究者对其进一步的审视。
- 我们提议临床研究报告应对此类审视开放，同时阐述了某制造商为拒绝提供我们完整临床研究报告所提供的差劲说辞。我们要求业界，要不就是开放临床研究报告，要不就要为其将随机临床试验视为机密的立场进行公开辩护。

Translation by Han-Pu Tung
